# The natural and human-mediated expansion of a human-commensal lizard into the fringes of Southeast Asia

**DOI:** 10.1186/s12862-024-02212-7

**Published:** 2024-02-20

**Authors:** Benjamin R. Karin, Michael Lough-Stevens, Te-En Lin, Sean B. Reilly, Anthony J. Barley, Indraneil Das, Djoko T. Iskandar, Evy Arida, Todd R. Jackman, Jimmy A. McGuire, Aaron M. Bauer

**Affiliations:** 1 Museum of Vertebrate Zoology and Department of Integrative Biology, University of California, Berkeley, CA 94720 USA; 2 Department of Biology, Villanova University, Villanova, PA 19085 USA; 3 Molecular and Computational Biology, University of Southern California, Los Angeles, CA USA; 4 Endemic Species Research Institute, 1, Minsheng E Rd., Jiji Township, Nantou County, 55244 Taiwan; 5 Department of Ecology and Evolutionary Biology, University of California, Santa Cruz, CA 95060 USA; 6 Department of Evolution and Ecology, University of California, 2320 Storer Hall, Davis, CA 95616 USA; 7 Institute of Biodiversity and Environmental Conservation, Universiti Malaysia Sarawak, 94300 Kota Samarahan, Sarawak Malaysia; 8 School of Life Sciences and Technology, Bandung Institute of Technology, 10 Jalan Ganesa, Bandung, 40132 Indonesia; 9 Basic Sciences Commission, Indonesian Academy of Sciences, 11 Jalan Medan Merdeka Selatan, Jakarta, 10110 Indonesia; 10 Research Center for Ecology and Ethnobiology, Badan Riset dan Inovasi Nasional (BRIN), Cibinong Science Center, Jalan Raya Jakarta-Bogor km 46, Cibinong, 16911 Indonesia

**Keywords:** Phylogeography, Biogeography, Invasive species, Scincidae, Southeast Asia, *Eutropis multifasciata*

## Abstract

**Background:**

Human-commensal species often display deep ancestral genetic structure within their native range and founder-effects and/or evidence of multiple introductions and admixture in newly established areas. We investigated the phylogeography of *Eutropis multifasciata*, an abundant human-commensal scincid lizard that occurs across Southeast Asia, to determine the extent of its native range and to assess the sources and signatures of human introduction outside of the native range. We sequenced over 350 samples of *E. multifasciata* for the mitochondrial ND2 gene and reanalyzed a previous RADseq population genetic dataset in a phylogenetic framework.

**Results:**

Nuclear and mitochondrial trees are concordant and show that *E. multifasciata* has retained high levels of genetic structure across Southeast Asia despite being frequently moved by humans. Lineage boundaries in the native range roughly correspond to several major biogeographic barriers, including Wallace’s Line and the Isthmus of Kra. Islands at the outer fringe of the range show evidence of founder-effects and multiple introductions.

**Conclusions:**

Most of enormous range of *E. multifasciata* across Southeast Asia is native and it only displays signs of human-introduction or recent expansion along the eastern and northern fringe of its range. There were at least three events of human-introductions to Taiwan and offshore islands, and several oceanic islands in eastern Indonesia show a similar pattern. In Myanmar and Hainan, there is a founder-effect consistent with post-warming expansion after the last glacial maxima or human introduction.

**Supplementary Information:**

The online version contains supplementary material available at 10.1186/s12862-024-02212-7.

## Introduction

Determining the extent of a species’ native versus introduced range is often difficult due to lack of records and extreme human modification to landscapes and transportation [1, 2]. Of these so-called cryptogenic species, widespread human-commensals that are good at dispersing both naturally and through human-movements are particularly difficult to decipher [3]. As the introduction of these species can lead to cascading ecological consequences, understanding the native range of species is important to conservation [4]. Phylogeographic and population genetic methods are critical tools to assessing if populations are native versus introduced, but they require comprehensive sampling of range-wide genetic diversity, and different invasion scenarios can lead to very different resulting patterns [5–7]. While single introductions to new areas show a classical founder-effect of extremely low genetic diversity in the new populations, multiple introductions are likely to instead produce high genetic diversity with divergent mitochondrial haplotypes in close proximity and admixture in nuclear DNA [5, 8–11]. Gene flow between introduced divergent lineages can restore genetic diversity to bottlenecked populations and provide the basis for adaptation that can worsen invasions [12–14]. Inside the native range, however, genetic structure is often preserved due to a combination of evolutionary and ecological processes [15–17]. Together these patterns can be used to understand the geographic extent of the native range and important biogeographic forces within it, and to pinpoint introduced populations.

The Many-lined Sun Skink, *Eutropis multifasciata* (Kuhl, 1820), is perhaps the most-commonly encountered diurnal lizard in Southeast Asia and possesses an incredibly large range from Bangladesh [18] through Indonesia [19]. It is an abundant inhabitant of urban areas, human-settlements, and forest edges, and is found from sea level to 1800 m in tropical areas [20] and 500 m at its northern extent in Taiwan [21]. Due to its abundance and relatively simple maintenance in a lab, it has become a model system for studying niche plasticity and thermal physiology, including its relationship with viviparity [21–27]. There are documented introductions to Florida [28, 29], Australia [30], New Guinea [31], and Taiwan (including nearby Ludao and Lanyu) [32, 33] due to frequent human-transportation including reports on airport luggage [34], ferries (J. McGuire, pers. obsv.), and international cargo shipments [35]. On Taiwan, Ludao, and Lanyu, *E. multifasciata* rapidly invaded and poses a significant conservation threat [32, 36, 37]. We used a geographically comprehensive genetic dataset of *E. multifasciata* to investigate its biogeographic history, the extent of its invasion, and the processes causing phylogeographic structure to be maintained or eroded. Together, these results can be utilized for conservation management in areas that have been recently invaded.

The phylogeography of *E. multifasciata* has been studied to some extent but still remains poorly understood. Barley et al. [38] investigated the population genomics of *E. multifasciata* with RADseq data, demonstrating that *E. multifasciata* has moderate levels of genome-wide genetic variation. Barley et al. observed higher genetic differentiation on islands relative to the mainland, recovering three Philippine lineages (southern; central/west; northern) and two weakly-differentiated continental populations (Myanmar; remainder of Indochina). They included only a few samples from Sundaland and Wallacea, limiting the scope of the phylogeographic analysis. The Philippine populations could be allied with Borneo, Sulawesi, or Indochina, or divided indicating multiple invasions of the Philippines [39]. Due to its random nature, RADseq data may encompass signatures of local adaptation, and this may be relevant when introduced populations have divergent environmental conditions to the source. For our study, the use of mitochondrial data may provide unique power to elucidate spatial patterns. The genome-wide data provides an interesting backdrop for our study, allowing us to understand the fine-scale mitochondrial structure we uncover given rates of nuclear gene flow estimated by Barley et al.

Since *E. multifasciata* is found in four major biogeographic regions: Indochina, Sundaland, the Philippines, and Wallacea [40], it is an interesting system to investigate how it is impacted by barriers that restrict many other species. We investigate if these regions are part of the native range, and if so, is there genetic structuring associated with the common biogeographic barriers that separate them? Mabuyine skinks are excellent dispersers that have crossed expansive marine barriers to distant islands [41–43], leaving the relative roles of natural vs. human-mediated dispersal in *E. multifasciata* an open question. An early phylogenetic study that included a few *E. multifasciata* samples recovered two major clades: one from Borneo, Myanmar, and China, and another from Java, Bali, Seram, and the Philippines [44], providing some indication that clades in *E. multifasciata* may not follow standard biogeographic area predictions (i.e. the Sunda Shelf not forming a clade). In another study, genetic structure across the Lesser Sunda Archipelago was substantial enough to reflect natural dispersal, and a clade on Bali and Timor was divergent from the remaining Lesser Sundas [19].

With comprehensive geographic sampling from over 350 samples, we investigated the biogeographic history of *E. multifasciata* to understand the extent of its native range and assessed the sources and signatures of human introduction outside the native range. We found it possesses a large native range where it has retained substantial genetic structure and test if this structure matches common biogeographic barriers or if it was eroded by human impacts. The well-documented invasion of *E. multifasciata* into Taiwan and neighboring islands provides a unique case-study to understand the signatures of human-mediated dispersal and assess whether expansion into other areas has been natural or human-mediated. We reanalyzed the RADseq data of Barley et al. [38] in this new context to better understand how ancestral mitochondrial structure can hold up to long periods of gene flow.

## Materials and methods

### Phylogenetic analyses

We used two datasets for phylogenetic analysis: 1) a mitochondrial ND2 dataset of 1023 bp with 361 samples of *Eutropis multifasciata* (343 were newly sequenced for this study; Supplemental Table S1) and 34 outgroups of *Eutropis*; and (2) a concatenated RADseq dataset comprised of 182,266 bp from 61 individuals (primarily from the Philippines). Only 18 individuals are common to both datasets, but geographically similar representatives are included from many of the clades. All newly generated sequences are deposited on GenBank (Supplemental Table S1).

For the ND2 dataset, trees were constructed using Bayesian Inference (BI) in MrBayes v3.2.1 [45], Maximum Likelihood (ML) in RAxML v8.1.15 [46], and divergence times estimated in BEAST v1.10.4 [47]. The appropriate partitioning scheme for each analysis was determined using PartitionFinder v2.1.1 [48] based on the Bayesian Information Criterion and greedy algorithm [49] and a starting tree using PhyML [50]. For each analysis, PartitionFinder specified a separate partition and substitution model for each codon position of ND2. For the ML analysis, a GTRCAT model was applied to each codon position separately and run with 1000 rapid bootstrap replicates. For MrBayes, the Markov Chain Monte Carlo (MCMC) was run for 100 million generations, with 4 chains, with the first codon position under GTR + I + G, the second under HKY + I + G, and the third under GTR + G. For all Bayesian analyses, we used Tracer v1.7.1 [51] to assess adequate convergence of the Markov chains and ESS values, and discarded the first 25% of trees as burn-in.

We reanalyzed the RADseq dataset of Barley et al. [38] in a phylogenetic framework, as it had only been previously analyzed specifically to investigate population and landscape genetics. We reduced the dataset to one individual per population for a total of 61 individuals to reduce the amount of missing data. Sequences were aligned using ipyrad v0.7.28, largely using default parameters except for clust_threshold = 0.94 and min_samples_locus = 46. The concatenated alignment consisted of 182,266 bp of sequence data containing 2928 parsimony informative sites. We estimated the phylogeny using four different programs: RAxML, MrBayes, BEAST, and SVDquartets [52] each producing a nearly identical topology. RAxML was run using the concatenated, unpartitioned alignment under the GTRCAT model with automatic rapid bootstrap termination. Two independent runs of MrBayes used the same alignment and were run under the GTR + G model with four chains and a temperature of 0.05 for 10 million generations sampling every 1000. The runs were combined and the first 25% of the trees were discarded as burnin. For SVDquartets, we randomly took one informative SNP per site for a total of 2392 SNPs. SVDquartets was run using PAUP v4.0a164 [53] with 100 bootstrap replicates to assess support.

### Divergence times

For the BEAST mitochondrial analysis, we removed the outgroups and analyzed the resulting 361 taxa alignment using a coalescent constant population size tree prior. The HKY + G model was chosen for the first and second codon positions and GTR + G was chosen for the third. We calibrated an uncorrelated lognormal molecular clock with a normally distributed prior with a mean of 0.00895 and standard deviation of 0.0025 [54]. The MCMC was run for 100 million generations and the first 25% of trees were discarded as burn-in.

Estimates of divergence times for phylogenetic analysis of RADseq data are difficult to accurately determine without appropriate fossil or secondary calibrations [55]. Still, we wanted to generate approximate divergence time estimates for the nuclear data for comparison with the mitochondrial estimates. We used BEAST v1.10.4 to estimate divergence times using a fixed, strict molecular clock set at 0.001 substitutions per site per million years, which was empirically derived from calibrations in other skink lizards [56]. We used a coalescent, constant population size tree prior, and modeled evolution of the concatenated matrix under the HKY + I + G model. The MCMC was run for 100 million generations sampling every 10,000 with convergence assessed as above. This molecular clock calibration is unlikely to be an accurate measure of the genome-wide substation rates across RADseq loci, however we use it to give an approximation of divergence times and to compare with the mitochondrial calibration.

### Species distribution model

We built a species distribution model (SDM) projected onto the last glacial maximum (LGM) to assess the extent that Pleistocene climatic fluctuations may have influenced relatively recent range expansions. This strategy assumes conservatism in the fundamental niche across climatic fluctuations, which may not have been the case especially in high latitude or high elevation regions and may underestimate the LGM distribution. We constructed the SDM with MaxEnt v.3.4.4 [57]. We combined our specimen localities with verified occurrence records from GBIF (accessed 13 Feb 2019; including museum records and iNaturalist research-grade observations). We reduced GBIF localities to those with relatively high precision and only took one record per unique latitude/longitude resulting in 1300 unique localities. To further reduce spatial auto-correlation, we reduced these localities to one point per 100 km using the *machu.occ.rarefy* command within the *machuruku* R package [58], resulting in 234 unique points. We downloaded 19 bioclimatic variables at 30 arcsecond resolution from the PaleoClim database [59] for the present and last glacial maximum (LGM) and cropped them to an extent from − 12° to 28° latitude and 88° to 141° longitude to include the entire range of *E. multifasciata*. We removed highly correlated variables by extracting pixel values at the occurrence points using the *extract* command in the *Raster* package [60] in R and testing for correlations. We reduced the set of variables to eight: Bio2 — Mean Diurnal Range, Bio3 — Isothermality, Bio4 — Temperature Seasonality, Bio5 — Max Temperature of the Warmest Month, Bio6 — Min Temperature of Coldest Month, Bio15 — Precipitation Seasonality, Bio16 — Precipitation of Wettest Quarter, Bio17 — Precipitation of Driest Quarter. We tested for the optimal set of feature classes and regularization multipliers using the *ENMevaluate* package in R, allowing for any combination of linear, quadratic, hinge, and product feature classes and regularization multipliers from 0.5 to 5 (by 0.5), 10, or 20. We used both linear and quadratic feature classes and selected a regularization multiplier of 3.5 based on the AUC. We ran the MaxEnt model using cross-validation for 10 replicates using a random 50% of the rarefied points for training and the remainder for testing. The median result of the replicates was summarized and plotted using QGIS [61] by selecting output raster values greater than 0.2.

## Results

### Phylogeography

**Fig. 1:**
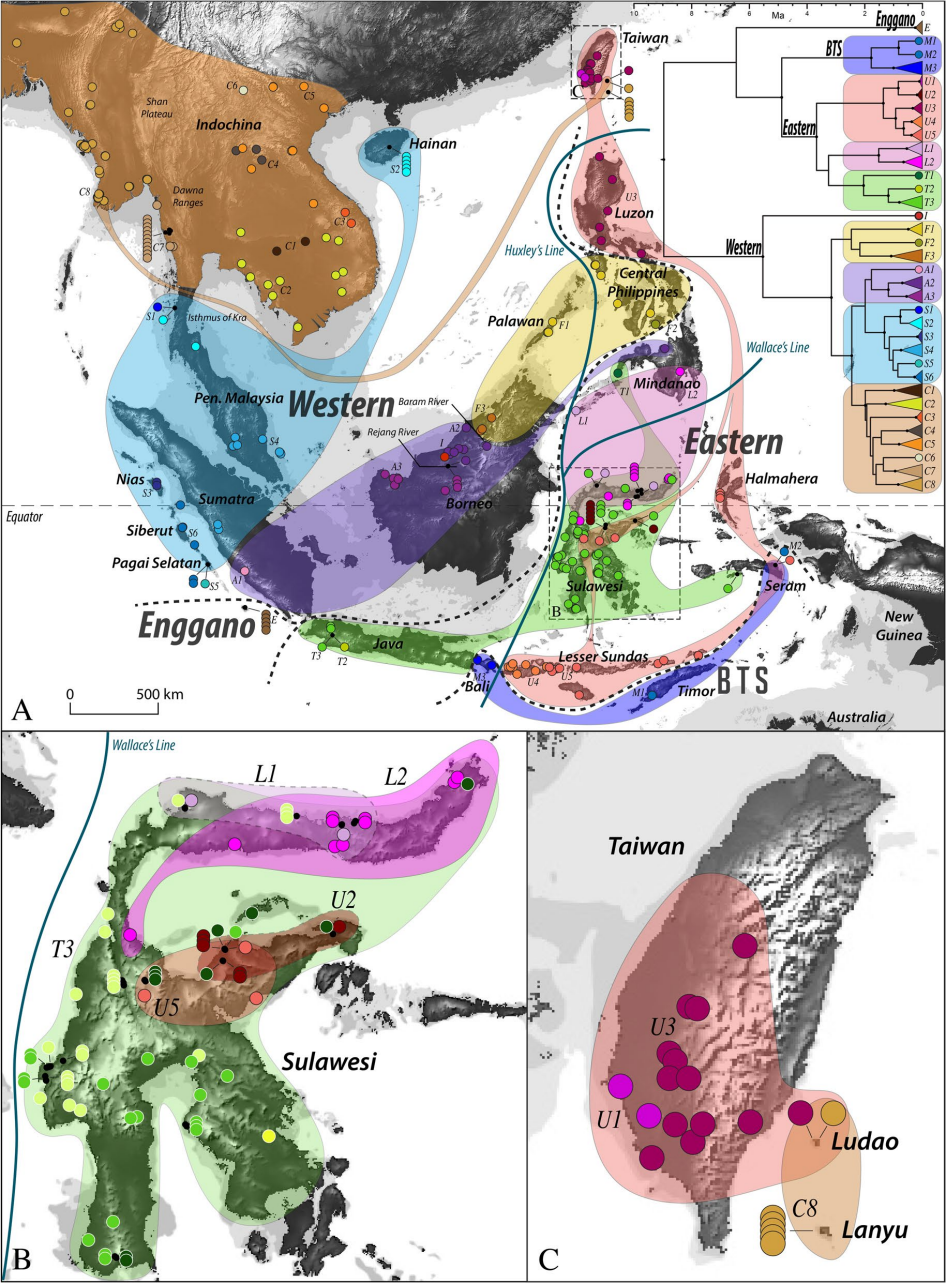
**A** Map displaying phylogeographic structure within *Eutropis multifasciata*, with the inset showing the mitochondrial BEAST timetree. Triangular tips in the tree have been collapsed and indicate more than one sample within a clade. The colors of geographic sampling points correspond to the tip colors in the tree and are nested according to the larger colored shapes corresponding to the encircled colored clades in the tree. Subclades are labeled corresponding to the tree tip labels. Nodes in the timetree with posterior probability greater than 0.95 are shown with a black dot, and timescale is in units of million years. Relevant biogeographic features discussed in the text are labeled. The dotted black lines indicate the geographic extent of the major numbered clades. Wallace’s Line and Huxley’s modification are shown in light blue for reference. The underlying map is shaded according to a digital elevation model (SRTM). The sea level bathymetry displays the 40 m (darker grey) and 120 m (lighter grey) depth contours (GEBCO), and indicate the areas exposed during periods of reduced sea levels. Location of inset maps shown by boxes. **B** Inset map of Sulawesi. White-rimmed points within the green T3 clade display additional phylogenetic substructure not indicated on the collapsed timetree. **C** Inset map of Taiwan, Ludao, and Lanyu

**Fig. 2:**
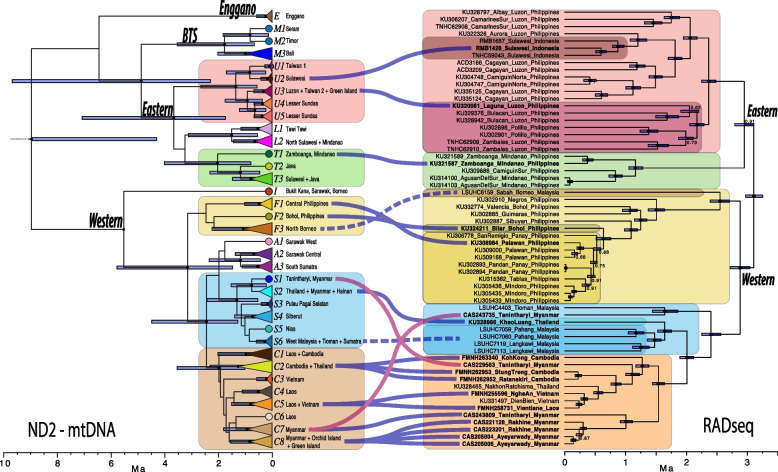
Comparison between the simplified ND2 BEAST tree (left) and the concatenated RADseq BEAST tree (right). Clades recovered in both datasets are indicated by colored boxes. Solid lines between the boxes represent samples that are present in both trees, whereas dashed lines represent clades that are presumed to be equivalent based on geographic location and tree position. The two magenta lines refer to instances of mismatch between the two trees and possible introgression. Black dots at nodes represent posterior probabilities greater than 0.95, or are labeled otherwise, and bars at nodes represent the 95% confidence interval on node height

We recovered four major mitochondrial clades within *E. multifasciata* in each analysis (Figs. 1 and 2) that are almost entirely geographically non-overlapping. (1) Western Clade — Borneo, Sumatra, Peninsular Malaysia, Indochina, Hainan, the central and western Philippines, Lanyu, and Ludao. (2) Eastern Clade — Java, Sulawesi, the Lesser Sunda Archipelago, the northern and southern Philippines, Taiwan, and Ludao. (3) BTS Clade — Bali, Timor, and Seram. (4) Enggano — Enggano Island. The mitochondrial and nuclear trees, though containing different sets of samples, showed nearly identical topologies with respect to the predominant subclades within the Western and Eastern Clades. The RADseq data only included representatives from the mitochondrial Western and Eastern Clades and not the BTS and Enggano Clades. Two samples showed mito-nuclear discordance between subclades separated by the Isthmus of Kra (Fig. 2; more details below).

The Western and Eastern Clades displayed different patterns: Western Clade subclades did not overlap geographically while several Eastern Clade subclades overlapped on individual islands. The boundary between Western and Eastern Clades in both the mitochondrial and nuclear data roughly follows Huxley’s modification of Wallace’s Line (see Fig. 1); however, Java and Bali on the Sunda Shelf are recovered within the Eastern and BTS clades and the central Philippines are recovered within the Western Clade.

We observed discordance with respect to the root position across the mitochondrial phylogenetic analyses. Using RAxML and MrBayes, we consistently recovered a poorly-supported topology of the BTS Clade sister to all remaining *E. multifasciata*, and Enggano sister to the Eastern Clade, whereas the BEAST molecular-clock rooting recovered the Western Clade sister to the remaining three clades with strong support (Fig. 1). Given this uncertainty, we do not assume any particular root position in the following phylogeographic discussion and instead focus on patterns within the major divergent clades themselves. See Supplementary Text for further details.

### Western clade

We recovered four primary subclades within the Western Clade that were geographically separated, and a fifth divergent outlier lineage. The boundaries between these subclades correspond to the Isthmus of Kra, between north and south Sumatra, and between north Borneo and the rest of Borneo. Interestingly, two samples from southern Myanmar (Tanintharyi) were recovered in discordant mitochondrial and nuclear clades across the Isthmus of Kra (see Fig. 2). One sample was recovered in the primarily west Malaysian mitochondrial clade (S1), but the nuclear Indochina clade, and the other sample shows the opposite pattern (C7). Within Indochina, we observed moderate genetic structure with more than eight well-supported slightly diverged mitochondrial lineages but low support for their relative relationships (C1–8). Peninsular Malaysia and offshore islands were recovered in a clade with central Sumatra and islands off the west coast of Sumatra, though with low support for their respective relationships (Fig. 1A, S1–6). Interestingly, samples from Hainan Island are recovered within this clade (S2), closest to southern Thailand and southern Myanmar (S1 and S2). In both RADseq and mtDNA data, northern Borneo (F3) was most closely related to the central Philippines (F1 and F2). A single sample from Bukit Kana in Borneo (I) was recovered as a divergent sister lineage to the entire remaining Western Clade. This single sample also possesses a divergent haplotype in four nuclear genes [62] (results not shown). See Supplementary Text for more details.

### Eastern clade

We recovered three primary subclades within the Eastern Clade: (1) A widely-distributed clade in the Lesser Sundas, Luzon, the Eastern Peninsula of Sulawesi, Seram, and Halmahera (U1–5). This clade also includes samples from Taiwan and Ludao; (2) The Northern Peninsula of Sulawesi, Mindanao, and Tawi Tawi in the Sulu Archipelago of the Philippines (L1–2); (3) A clade spanning almost all of Sulawesi but also with representatives on Java, the Zamboanga peninsula of Mindanao, and Ambon (T1–3). Sulawesi was found to contain representatives of all three of the above subclades, and had substructure within each of them (Fig. 1B). The eastern Lesser Sundas allied with Seram, Halmahera and Sulawesi. Luzon (U3) was sister to the two primarily Lesser Sunda clades (U4 and U5). The predominant lineage on Taiwan was identical to haplotypes on Luzon (U3), and was also found on Ludao (Fig. 1C). See Supplementary Text for more details.

### BTS and Enggano clades

The BTS Clade was comprised of three divergent lineages spanning across Wallace’s Line. A sample from Timor (M1) was recovered with strong support as sister to a sample from Seram (M2). Several samples from Bali (M3) were recovered as sister to Timor and Seram together. The BTS Clade was 5–9% divergent (raw-pairwise divergence) from all remaining *E. multifasciata*. The Enggano Clade was comprised solely of samples from remote Enggano island. This clade was also 5–9% divergent from all remaining *E. multifasciata*.

### Divergence times

The mtDNA and RADseq divergence time estimates differed substantially (median crown age of mtDNA: 9.0 Ma; RADseq: 3.1 Ma; see Fig. 2). We emphasize that these molecular clock calibrations should be interpreted with significant caution. We chose to include the divergence time estimates only to show a broad range of possible ages of these clades, rather than to test specific dispersal timing hypotheses. It is difficult to accurately estimate divergence times using RADseq data without fossil or secondary calibrations [55] that are unavailable for *Eutropis* [41]. In the RADseq data, coalescence times between the Western vs. Eastern clades are only about 7500 generations old and migration rates (in units of 2N_e_m) range from 0.05–4 per generation [38]. This highlights that moderate amounts of gene flow are present across the range of *E. multifasciata*, and that a bifurcating tree is an inaccurate representation of the reticulating intraspecific genealogy. Our estimated nuclear divergence times therefore only represent a rough indication of overall genetic divergence at our assumed clock rate and are likely biased towards older divergence estimates. Despite these limitations, the overall topological concordance between the mitochondrial and nuclear clades indicates that the mitochondrial tree is still likely to be a good indicator of the original dispersal pathways.

### Species distribution model

**Fig. 3:**
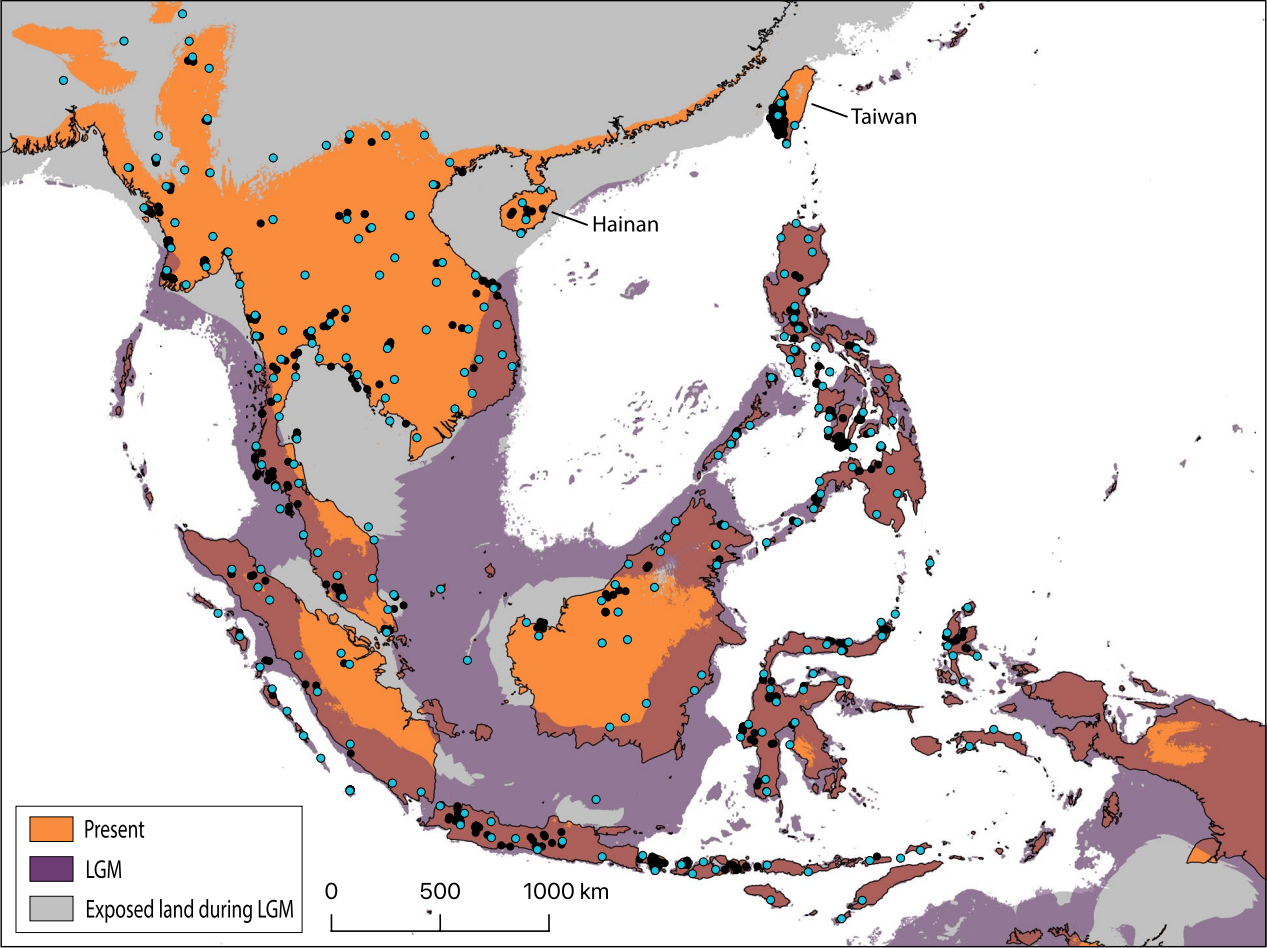
Simplified species distribution model for *Eutropis multifasciata* under the present climate (orange) and projected on the climate of the last glacial maximum (LGM; Purple). Much of its northern range is likely to have been uninhabitable during the LGM. Back points indicate all georeferenced localities of *E. multifasciata*, and blue points indicate the rarefied localities used to train and test the species distribution model to avoid spatial autocorrelation. Note that all of Hainan and most of Taiwan are predicted to have been uninhabitable during the LGM

The SDM predicted areas occupied by *E. multifasciata* (Fig. 3)*.* The ten replicates had an average area under curve of 0.766 and standard deviation is 0.034. Bio2 — Mean Diurnal Range had the highest percent contribution (41.9%), followed by Bio6 — Min Temperature of Coldest Month (37.9%). Bio2 also had the highest permutation importance (65.6%), followed by Bio4 — Temperature Seasonality (26.1%). The projected LGM SDM suggests the range of *E. multifasciata* contracted substantially, with much less suitable habitat especially in the northern part of its range. Hainan and all but the southern tip of Taiwan are predicted to have been uninhabitable during the LGM. Some interior regions of the exposed Sunda Shelf were unsuitable, though suitable corridors are predicted. All islands in the Philippines and Wallacea are predicted to have been suitable.

## Discussion

### Phylogeography

Despite being frequently transported by humans, human-commensal species most often retain ancestral genetic structure across their native range whereas newly introduced populations show classical founder-effects or evidence of multiple introductions [5, 8, 9, 11, 63–65]. In this study we find evidence that *E. multifasciata* naturally colonized most of its enormous range across Southeast Asia where it retains substantial mitochondrial and nuclear genetic structuring, and that only the northern and eastern fringes were the result of recent human introduction or post-warming expansion.

### Western clade — mitochondrial breaks correspond to expected biogeographic barriers

Within the Western Clade, several of the major genetic breaks correspond to commonly observed biogeographic barriers in other taxa, suggesting it is all or nearly all part of the native range. The major break within *E. multifasciata* in mainland southeast Asia and the Sunda Shelf occurs near the narrow Isthmus of Kra (Clades C and S). The Isthmus of Kra is a major biogeographic barrier separating the biogeographic regions of Sundaland and Indochina [66]. It is common for population or species turnover to occur north or south of the narrowest part of the isthmus [67]. The observed discordance across the isthmus between mitochondrial and nuclear data (Fig. 2) indicates that there is gene flow between the subclades, even at this substantial barrier for most taxa. This is not surprising given the dispersal capability of *E. multifasciata* and suggests this is a porous barrier. Gene flow can produce unusual artefactual phylogenetic patterns [68–70], so a careful assessment of these lineage boundaries beyond the mitochondrial patterns is warranted.

The genetic breaks we found on the Sunda Shelf also correspond to several frequently observed biogeographic barriers. Peninsular Malaysia and central Sumatra were recovered together (S4), matching a commonly observed pattern as they are only separated by a narrow channel and have been frequently connected by land during Pleistocene sea-level fluctuations [71–73]. The connection between southern Sumatra and northwestern Borneo (though poorly supported in the analyses) also has been previously reported and likely reflects their previous connection by a raised land-corridor [13, 71, 74]. The main split in northern Borneo (between clades A and F) is also a commonly observed pattern. In several bird species, populations in northwestern Borneo are more closely related to those in eastern Sumatra or Peninsular Malaysia than north Borneo [75–79]. The LGM projection of the species distribution model is consistent with the existence of dispersal corridors for *E. multifasciata* across the exposed Sunda Shelf (Fig. 3). The exposed Sunda Shelf is predicted to have been a more xeric, savannah-like environment during these climatic fluctuations [80–83], so it is possible that species such as *E. multifasciata* that are adapted to open and disturbed habitats were more suited to crossing it than rainforest-adapted species, such as the congener *Eutropis rugifera*, that faced barriers [84].

Across northern Borneo, where we have the finest-scale sampling, our results support the large Rejang and Baram rivers as barriers separating the mitochondrial lineages. The split on Sumatra is also concordant with the location of the large Batang Hari river, though sampling is not sufficient to confirm the specific location. It is counter-intuitive that freshwater rivers would act as such strong genetic barriers after considering how the species was able to cross much more substantial marine barriers. Trans-riverine dispersal should be more frequent and bidirectional while trans-marine should be rarer and generally unidirectional. Under the assumption that there exists a genetic priority effect preventing or reducing gene flow between clades [16], then these barriers likely reflect a geographic weakness or habitat break where a tension zone can settle on rather than a strong isolating force [85].

Further north, we observed a genetic break between east and west lineages across Indochina in the nuclear data that was poorly supported by the mtDNA. These lineages diverged recently (435 generations) and show strong unidirectional gene flow from east to west (3.99 effective migrants per generation) [38]. The Shan Plateau and Dawna ranges between Myanmar and Thailand may be difficult for *E. multifasciata* to cross, potentially reinforcing the break in this area. Furthermore, our LGM SDM (Fig. 3) suggests that *E. multifasciata* in Indochina was split along Indochina’s eastern and western coasts during global cooling events (though this may be an underestimate), and the post-warming expansion northwards could have brought these moderately divergent clades back into contact along a natural barrier. India, Bangladesh, and much of Myanmar were likely colonized during a Pleistocene post-warming range expansion as these populations show very low genetic diversity (also see [18]). In Thailand, Cambodia, Laos, and Vietnam, we do not observe mtDNA phylogeographic structuring associated with clear geographic barriers, but the clades are generally nonoverlapping indicating that some geographic structuring is being maintained in this region.

The mtDNA and nuclear data both split the Philippines into three separate clades: Mindanao, Luzon, and the Central Philippines (Fig. 2; also see [38]). These results suggest that Palawan was a stepping-stone pathway for dispersal into the central Philippines, as has been proposed for several taxa [39, 86]. Divergence times between Borneo and the central Philippines occurred after the Philippines was near its present geographic conformation [39]. There is a moderate amount of gene flow between the Central Philippines and both Mindanao and Luzon (1.7–2.1 effective migrants per generation) [38], though clearly not enough to erode the phylogeographic structure we observed. It is unclear why the Philippine clades are divided as such, but it could be through replacement of one clade by another and/or selective processes leading some populations to be better adapted to small versus large islands [17]. In any case, given the high levels of genetic diversity concordant with geography, the Philippines are very likely a part of the native range.

### The Bukit kana outlier

It is unclear what process could lead a single sample from Bukit Kana (I) in Sarawak on Borneo to be recovered, on its own, sister to all remaining Western Clade samples. The first author sequenced four nuclear genes for this sample for his Master’s thesis, and found it held a divergent haplotype for two genes, but was very similar to Sarawak samples in the other two genes [62]. This positioning could indicate hybridization between representatives of the Eastern and Western clades [13, 68–70], though there was no evidence of increased heterozygosity in these markers, suggesting it is not a recent hybrid. What is puzzling about the placement in the mtDNA tree is that we should not observe an intermediate hybridization tree placement effect in mtDNA as it is uniparental. This raises the possibility that this haplotype represents a relict mitochondrial lineage that has held up to subsequent nuclear gene flow. It will be interesting to test these alternatives in future work with increased sampling in this region.

### Java does not follow Wallace’s line

The only location where Clade 2 crosses to the west side of Wallace’s Line is Java, where most samples were nested in a clade comprised predominately of Sulawesi samples (T3), but we also observe another haplotype present (T2). Despite Java’s proximity to Sumatra and Borneo, species and populations on Java are often found to be divergent from the remaining Sunda Shelf and closely related to Wallacean counterparts [87]. However, since Java has the highest human population density of any island in the world, it is conceivable that on Java the ancestral genetic structure may have been overridden by human-introduction from the increased travel and shipping from neighboring areas. In an early study with just a handful of samples and different genetic markers, Java was recovered with Bali, Seram, and the Philippines [44], so the BTS clade could be an ancestral mitochondrial lineage on Java.

### Eastern clade — complex phylogeography and signatures of multiple introductions

Patterns in the Eastern Clade contrast starkly with those in the Western Clade, with many of the major subclades overlapping geographically, which is likely the result of multiple human introductions. Multiple introductions will often lead to haplotypes from multiple sources in a single new population leaving behind a potentially misleading signature of high genetic diversity [5, 9, 88]. Sulawesi stands out in holding five divergent mitochondrial lineages. Mindanao holds three, Seram two, and Taiwan two. We consider at least some of these cases to be the result of human-mediated dispersal (and especially after considering Taiwan, discussed later), as these clades would be unlikely to come into sympatry otherwise.

Some of the structure on Sulawesi is likely the result of the biogeographic history of the island, though it appears to have been eroded to some extent. Even as recent as 2 Ma, Sulawesi was divided into seven or more paleoislands [89] on which lineages of many species have been shown to diverge substantially [90]. Our results are consistent with isolated lineages of *E. multifasciata* on these paleoislands coming into secondary contact as these paleo-islands fused to form the present-day Sulawesi. For example, lineages L1 and L2 may have diverged on the paleoislands making up the northern Peninsula, and subsequently come into contact (Fig. 1B). It is intriguing that priority effects that seem to be strong in other parts of the range do not appear to be keeping these lineages from becoming sympatric. Perhaps it reflects the divergence that accumulated between populations, which may not have been substantial enough to prevent nuclear gene flow and segregation of mitochondrial haplotypes. Nevertheless, our results are consistent with Sulawesi as a source of lineage diversity that possibly expanded from there into other areas, including the Philippines, Java, and eastern Wallacea.

### BTS clade — a relict or human-mediated?

The BTS Clade has been previously recovered [19], but without broader geographic sampling to assess its evolutionary history. It is unusual to see an association between these islands and not the other intermediate Lesser Sunda islands [19, 91, 92], and there is no clear biogeographic explanation for this result. It does show some similarity to *Cyrtodactylus* which had multiple waves of dispersal through the Lesser Sundas [93]. One possible scenario is that the BTS Clade was more widely prevalent across the Lesser Sunda Archipelago but was later replaced and relictualized by the Eastern Clade. The substantial molecular divergences in this clade reject human-mediated dispersal. This clade is divergent but should not necessarily be taxonomically recognized. A Bali subspecies (*E. m. balinensis*) was previously recognized, but was synonymized after a morphological comparison found no significant differences [94].

### Enggano clade — an ancient, isolated lineage

We recovered a divergent outlying lineage only including specimens from Enggano Island. Enggano is an isolated deep-water island 120 km off the coast of Sumatra that hosts several endemic lizards including two geckos and a flying lizard, and future molecular studies are likely to uncover new endemics [95]. Within the range of *E. multifasciata*, Enggano is one of the most isolated islands in terms of distance from other possible sources, yet for a species with such high dispersal capability it is still surprising to find such a divergent lineage here and not on several of the other isolated islands it occupies. Further investigation of morphological and nuclear genetic divergence will assess if this population warrants taxonomic recognition as a subspecies or species.

### Recent natural and human-mediated expansion at the fringe

The well-documented invasion of *E. multifasciata* into Taiwan, Ludao, and Lanyu provides a unique opportunity to understand the signature of human-mediated dispersal and allows us to assess other candidate cases by looking for similar patterns. In 1992, *E. multifasciata* was first documented in Taiwan near the Kaoshiung international trading port [33], presumably from a cargo or timber shipment, and subsequently spread across much of the south and central parts of the island [32]. Today *E. multifasciata* has become very successful on Taiwan and only seems to be restricted from areas with cold winter temperatures [21]. Repeated Pleistocene cooling events expanded this unsuitable area to nearly all of Taiwan (Fig. 3), and this is likely why *E. multifasciata* did not establish a persistent natural population despite movement of early humans between Taiwan and the Philippines [96]. In 2008, *E. multifasciata* was discovered on Ludao near the international airport with a biased sex ratio indicating it recently colonized the island [37]. Since then it has remained isolated to an area about 10 ha around the airport and has been the subject of a significant eradication effort [36, 37]. The arrival on Lanyu is less clear, and it has been questioned whether it was natural or human-mediated [32, 97].

Our results suggest multiple human introductions to Taiwan and offshore islands, some of which have clear sources. Most of Taiwan shares a haplotype with Luzon (U3). Luzon is the closest natural population of *E. multifasciata* to Taiwan with a major port, and it could have easily been carried by a cargo or timber ship. This haplotype is also represented by one sample from Ludao, indicating a potential introduction from mainland Taiwan or separately from Luzon to the island. A second introduction is from a unique but closely related clade represented by two samples from southwest Taiwan (U1). The second source is less clear, as the samples are resolved sister a population on Sulawesi that could be natural or introduced (U2). The third unique haplotype is present on Lanyu and Ludao and is shared with Ayeyarwady, Myanmar (C8). All samples from Lanyu possess this Myanmar haplotype, whereas only one sample from Ludao possesses it (all others from Ludao possess the Luzon haplotype, T. Lin, unpublished data). The tiny island of Ludao is the only location in the entire range where the Western and Eastern Clades occur in sympatry, a clear signature of two human introductions. Hybridization of these divergent lineages could provide genetic variation for which it could evolve traits for better invasion success, allowing it to spread to new areas [14].

We assessed other possible cases of human introduction by looking either for divergent haplotypes in sympatry or a founder-effect, and observed that all the possible cases occur at the northern and eastern fringes of what we expect is the native range. There are only a few islands other than Taiwan and Ludao with multiple divergent clades: Mindanao, Sulawesi, and Seram. Mindanao is represented by three divergent haplotypes, one nested within the clade in northern Borneo (A2), the second nested within the clade on the northern peninsula of Sulawesi (L2), and the third (T1) sister to the clade including Java and most of Sulawesi (T2 and T3). The first (A2) is likely human-introduced as it is nested within a northern Borneo clade and collected near an airport outside the highly urbanized city of Cagayan de Oro. The second lineage is also likely human-introduced as it shares a haplotype with Sulawesi samples and was collected in Davao City, the largest city on Mindanao. The third lineage (T1) could correspond to a natural dispersal, as it is a divergent mitochondrial haplotype and is shown to be more widespread across Mindanao from the RADseq data (Fig. 2). This is consistent with natural dispersal between Mindanao and Sulawesi along the Sangihe-Talaud Archipelago [39, 98], though the direction is unclear. Mindanao is a large and complex island that harbors seven species of *Eutropis* [84, 99] so multiple lineages may be more able to persist on different parts of this island than on others.

At the southeast edge of the range, the Lesser Sunda lineages likely represent a recent natural expansion [19], but one of the lineages (U5) may have been introduced to Seram, central Sulawesi, and Halmahera as these all share nearly identical haplotypes—evidence of a founder-effect. On Seram, we also observe multiple introductions, but since *E. multifasciata* has been on Seram for at least 100 years [100] a more thorough investigation is warranted to confirm human introduction for both haplotypes. Seram and Halmahera are at the very edge of the range, and close to New Guinea where *E. multifasciata* recently established another new population [31].

The observed low genetic diversity in at the northern edge of the range could possibly be the result of either post-warming expansion or human-introduction. The LGM SDM is concordant with Taiwan being mostly uninhabitable during that time, but Hainan and much of Indochina are also predicted as uninhabitable (Fig. 3). This is likely due to colder winter temperatures as this currently limits its elevational and latitudinal range in Taiwan [21] and is a top predictive variable in the SDM. Many refugia likely persisted across Indochina that could explain the observed high genetic diversity; however, northeastern India and northern Myanmar show a possible founder-effect consistent with post-warming expansion [15]. Similarly, all samples from Hainan share an identical haplotype, but interestingly it matches southern Thailand south of the Isthmus of Kra (S2) and this haplotype is closely related to nearby southern Myanmar (S1 and S2). If the Hainan population dispersed naturally, then this relationship is strange as we would expect the dispersal to come from a nearby landbridged Indochina clade. Further, if *E. multifasciata* had been present on Hainan since before the LGM and was able to persist in refugia (e.g., along the changing coastline), why then is it not recovered as a divergent lineage but is instead identical to the sample in Myanmar? The earliest museum specimen of *E. multifasciata* from Hainan was collected ca. 1909 (MCZ:HERP:R-7384, exact collection year not specified) and several more specimens were collected in 1923 at multiple localities (MCZ:HERP:R-39307–8; FMNH-7252–61), so the timing of potential human introduction would have been quite old. Still, Hainan’s treaty port at Haikou had been active since 1858 [101], so *E. multifasciata* could easily have been introduced from a trading ship in the late-1800’s. This hypothesis should be tested with nuclear genetic data from additional localities on Hainan to rule-out more recent mitochondrial introgression or natural expansion and to assess population demography across the island.

## Conclusions

Based on our results from Taiwan, Ludao, and Lanyu, and other instances of indisputable [29, 31, 34] and potential (Sulawesi, Mindanao, Seram, Halmahera) human-mediated dispersal, it is clear that *E. multifasciata* is very frequently transported around Southeast Asia. In the native range, as evidenced by genetic structure matching common biogeographic barriers, ancestral structure has held up to this frequent human-mediated dispersal. At the fringes, however, we observe divergent haplotypes in sympatry or founder-effects indicative of recent range expansion. Our results support *E. multifasciata* holding an enormous native range across much of Southeast Asia, and even as it became highly abundant in human-disturbed areas the observed ancient genetic structure remained intact. This work provides an approach that can be applied to other species to investigate their native vs. introduced ranges.

In the native range, we hypothesize that genetic structure is maintained through a combination of high-density blocking and/or local fitness advantages that could be the underlying basis of a genetic priority effect. If new populations become locally-adapted to their unique environment [9], which can evolve relatively rapidly from standing variation, they can hold back incoming gene flow from other areas [16, 102]. Local-adaptation may also buffer against climatic shifts, and could have led to underestimates of our LGM SDM and impacted our RADseq results. High-density blocking prevents new migrants from establishing and introgression from successfully occurring simply due to raw numbers of individuals and corresponding allele frequency differences [15, 103]. Combined, it may be very difficult for gene flow to erode existing phylogeographic structure. Contact between lineages may not initially coincide with biogeographic barriers, but may move and settle on even narrow habitat breaks such as those we observed across rivers, mountain ranges, and isthmuses [85]. Conversely, at the fringes of the native range, this pattern breaks down due to near-simultaneous introduction of skinks by humans to previously uninhabited islands before these processes are established.

Overall, the phylogeography of *E. multifasciata* is complex owing to its incredibly large natural range in one of the most biogeographically complex and dynamic regions of the world [40]. Within the native range, it showcases several classical biogeographic patterns (e.g., Isthmus of Kra) while also not conforming to others (e.g., Philippines). As such, deciphering the exact extent of its native range is difficult, but it is still clear that recent expansion has been primarily limited to the northern and eastern edges of its range. *E. multifasciata* has established small populations around the world through human introduction, but only in Taiwan and offshore islands at the edge of its native range have populations grown explosively. This contrasts with other invasive skinks, such as *Lampropholis delicata* and *Carlia ailanpalai*, which also display substantial phylogeographic structuring in their native ranges in Australia and New Guinea, but have become invasive across the Pacific and do not appear to undergo the same edge-restricted expansion [65, 88, 104]. Future work following-up on new populations with known or approximate dates of establishment will be an interesting comparison with our results.

### Supplementary Information

**Additional file 1:** **Supplementary Text.**

**Additional file 2:** **Figures S1-S8.**

**Additional file 3:** **Table S1.** Specimens used in phylogenetic analyses and corresponding GenBank accession numbers. Specimens at the top of the list are outgroups and followed by samples already deposited on GenBank. Published genetic sequences accessed from alternative repositories have the link available.

## Data Availability

All sequences are available on Genbank (Table S1) and full phylogenetic trees for all analyses can be found in the Supplemental Material.
