# Supplementary material for: The natural and human-mediated expansion of a human-commensal lizard into the fringes of Southeast Asia
Source: BMC Ecol Evol. 2024 Feb 20;24:25. doi: 10.1186/s12862-024-02212-7 (PMC10880348; doi:10.1186/s12862-024-02212-7)
Supplement: Supplementary file 1 — Additional file 1: Supplementary Text. [file 12862_2024_2212_MOESM1_ESM.pdf]

The natural and human-mediated expansion of a human-commensal lizard into the fringes of Southeast Asia

Benjamin R. Karin[1,2,*], Michael Lough-Stevens[2,3], Te-En Lin[4], Sean B. Reilly[1,5], Anthony J. Barley[6], Indraneil Das[7], Djoko T. Iskandar[8,9], Evy Arida[10], Todd R. Jackman[2], Jimmy A. McGuire[1,#], Aaron M. Bauer[2,#]

Supplementary Text

Supplemental Methods

For mtDNA sequencing, genomic DNA was extracted as described using a salt extraction method [1, with modifications]. Polymerase chain reactions (PCR) was performed on an Eppendorf Mastercycler nexus gradient thermocycler and PCR product was cleaned using a home-made magnetic bead solution [2]. All PCR reactions began with an initial 2 min denaturation at 95°C followed by 95°C for 35 s, annealing for 35 s at 50ºC, and extension at 72°C for 1 min 35 s for 34 cycles. Cycle sequencing was performed using Big Dye v3.1 chemistry, followed by an additional magnetic bead cleanup and analysis on an ABI3730xl. ND2 primers were MetF1 5'-AAGCTTTCGGGCCCATACC-3' [3] and CO1R1 5'-AGRGTGCCAATGTCTTTGTGRTT-3' [4].

We calculated pairwise genetic distances between all samples and their association with geographic distance, a classic way to visualize the effect of isolation by distance. We do not explicitly test for isolation by distance, as this has been done with more comprehensive sequencing by Barley et al. [5], however we simply use it as a useful and intuitive visualization tool. We calculated pairwise DNA distances in R using the *dist.dna* function in the *ape* package [6] under the TN93 model and specifying automatic deletion of missing data for each sample pair. We calculated pairwise geographic distances with the *distm* function in the *geosphere* package [7] in R under the *distGeo* function. We plotted all values, colored by clade, and then selectively removed certain clade comparisons for easier visualization. 95% ellipses were generated using *ggplot2* [8].

Supplementary Results

We observed discordance with respect to the root position across the mitochondrial phylogenetic analyses that were outgroup vs. time-rooted. When outgroups were included in the RAxML and MrBayes analyses, we consistently recovered a poorly-supported topology of the BTS Clade sister to all remaining *E. multifasciata*, and Enggano sister to the Eastern Clade (Supplementary Figs. S2–3). When we ran BEAST under a molecular-clock rooting strategy with or without an outgroup (BEAST results with outgroup not shown), we recovered the Western Clade sister to the remaining three clades with strong support (Fig. 1) (note that these differing rooted topologies are identical when unrooted). The BTS and Enggano Clades were not represented in the RADseq dataset. Given this uncertainty, we do not assume any particular root position in the phylogeographic discussion and instead focus on patterns within the major divergent clades themselves. All RADseq analyses consistently recovered the same higher-level clades as the mtDNA (Fig. 2), though the relationships between some of the tips within these clades were variable between analyses (Supplementary Figs. S4–7). Besides the rooting, the

different mitochondrial trees were nearly identical, and supported all the same well-supported lineages (Supplementary Figs. S1–3).

Western Clade

Within Indochina, we observed moderate genetic structure with more than eight well-supported slightly diverged mitochondrial lineages but low support for their relative relationships (C1–8). Two mtDNA lineages were recovered in northern (C8) vs. southern Myanmar (C7), and the remaining six lineages were spread across Thailand, Cambodia, Vietnam, and Laos and are mostly nonoverlapping geographically (except C4 and C5). Clade C8 was also found on Ludao and Lanyu near Taiwan, most closely related to samples from Ayeyarwady (Fig. 1C). Samples from Indochina also exhibited substantial nuclear genetic variation in the RADseq data, separating into two distinct clades: one in Myanmar and the other primarily in Thailand, Vietnam, Cambodia, and Laos (see Fig. 2). One sample from Tanintharyi, Myanmar was recovered in the latter nuclear clade.

Peninsular Malaysia and offshore islands were recovered in a clade with central Sumatra and islands off the west coast of Sumatra, though with low support for their respective relationships (Fig. 1A, S1–6). Within clade S4, western Peninsular Malaysia allied with Sumatra, whereas eastern Peninsular samples allied with Pulau Tioman and Pulau Tulai off the east coast. The islands off the west coast of Sumatra were recovered in three divergent subclades, one from Pulau Nias (S3), one from Pulau Sipura, Pulau Pini, Pulau Siberut, and Pulau Pagai Selatan (S6), and a final divergent single sample also found on Pulau Pagai Selatan (S5). Interestingly, samples from Hainan Island are recovered within this clade (S2), closest to southern Thailand and southern Myanmar (S1 and S2). Hainan samples shared an identical haplotype with one sample from southern Myanmar (S2).

In both RADseq and mtDNA data, northern Borneo (F3) was most closely related to the central Philippines (F1 and F2). In the mtDNA, Clade F was separated from Clade A across the Baram River in western Borneo. The remainder of northwestern Borneo (A2 and A3) allied with southern Sumatra (A1), though with poor support. Northwestern Borneo was divided into two closely-related sister clades (A2 and A3) corresponding to the Rejang River. A single sample from Bukit Kana in Borneo (I) was recovered as a divergent sister lineage to the entire remaining Western Clade. This single sample also possesses a divergent haplotype in four nuclear genes (Karin, 2016; results not shown).

Eastern Clade

We recovered three primary subclades within the Eastern Clade: (1) A widely-distributed clade in the Lesser Sundas, Luzon, the Eastern Peninsula of Sulawesi, Seram, and Halmahera (U1–5). This clade also includes samples from Taiwan and Ludao (discussed later); (2) The Northern Peninsula of Sulawesi, Mindanao, and Tawi Tawi in the Sulu Archipelago of the Philippines; (L1–2) (3) A clade spanning almost all of Sulawesi but also with representatives on Java, the Zamboanga peninsula of Mindanao, and Ambon (T1–3).

Sulawesi was found to contain representatives of all three of the above subclades, and had substructure within each of them (Fig. 1B). The northern peninsula of Sulawesi was divided between clades L1 and L2. Each of these had a close relative in the Philippines, with the small island of Tawi Tawi near Borneo (L1) and with Mindanao (L2).

Clade T3 was found across the entirety of Sulawesi and held a substantial amount of substructure. We recovered low support for the relationships among the substructured lineages, and they all overlapped geographically except a single divergent sample from the southeast peninsula (see Fig. 1B, white-rimmed points). Two other islands formed distinct and separate lineages within the T3 clade: Haruku and Java. Of a total of four Java samples, three were nested within T3 and the final sample (T2) was recovered sister to T3. A single sample from the Zamboanga Peninsula of Mindanao was recovered as sister to the combined T2 and T3 clade.

The eastern peninsula of Sulawesi was primarily occupied by two clades, one of which shared haplotypes with Sumba and Flores in the Lesser Sundas (U5) and the other was sister to two samples from Taiwan (U1). The Lesser Sundas were divided into western (U4) and eastern (U5) sister subclades meeting within the island of Sumbawa. The eastern Lesser Sundas allied with Seram, Halmahera and Sulawesi. Halmahera shared a haplotype with Flores, and Seram shared one with Sumbawa. The Northern Philippines island of Luzon (U3) was sister to the two primarily Lesser Sunda clades (U4 and U5). The predominant lineage on Taiwan was identical to haplotypes on Luzon (U3), and was also found on Ludao (Fig. 1C).

DNA distances

Pairwise DNA distances plotted against geographic distance and distances show two main clusters of points. The first cluster is within-clade, with distances of 0 to 5%. The second cluster is between the major clades, with most distances falling between 6 and 9%. Note that in the plot some comparisons were removed for easier visualization. Taiwan, including Ludao and Lanyu, displays the largest spread of genetic distances because it is the only location with representatives of Eastern and Western Clades in very close geographic proximity. A significant correlation with DNA distance and geographic distance has already been shown by Barley et al. (2015) using the RADseq data.

Supplementary Discussion

The discordance in the root position across analyses prevents us from confidently assessing where *Eutropis multifasciata* originated using ancestral area reconstructions. Still, we expect it to likely be on the Sunda Shelf, primarily because all of the main clades occur either directly on the Sunda Shelf (Western Clade: Borneo, Sumatra, Peninsular Malaysia; Eastern Clade: Java; BTS Clade: Bali) or in the case of the Enggano Clade on an offshore island near the Sunda Shelf. Further, the divergent single sample from Bukit Kana on Borneo may be a relict of a more widespread clade that was later replaced or could be a result of historic introgression between the Western and another Clade on the Sunda Shelf. Historic introgression as the clades initially diverged could also explain the observed discordance in rooting, though it could be the result of other issues such as insufficient phylogenetic signal or long branch attraction.

References

1. Aljanabi SM, Martinez I. Universal and rapid salt-extraction of high quality genomic DNA for PCR-based techniques. Nucleic Acids Res. 1997;25:4692–4693.
2. Rohland N, Reich D. Cost-effective, high-throughput DNA sequencing libraries for multiplexed target capture. Genome Res. 2012;22:939–946.

3. Macey JR, Larson A, Ananjeva NB, Fang Z, Papenfuss TJ. Two novel gene orders and the role of light-strand replication in rearrangement of the vertebrate mitochondrial genome. Mol Biol Evol. 1997;14:91–104.
4. Arevalo E, Davis SK, Sites JW. Mitochondrial DNA sequence divergence and phylogenetic relationships among eight chromosome races of the *Sceloporus grammicus* complex (Phrynosomatidae) in central Mexico. Syst Biol. 1994;43:387–418.
5. Barley AJ, Monnahan PJ, Thomson RC, Grismer LL, Brown RM. Sun skink landscape genomics: Assessing the roles of micro-evolutionary processes in shaping genetic and phenotypic diversity across a heterogeneous and fragmented landscape. Mol Ecol. 2015;24:1696–1712.
6. Paradis E, Claude J, Strimmer K. APE: Analyses of phylogenetics and evolution in R language. Bioinformatics. 2004;20:289–90.
7. Hijmans R, Williams E, Vennes C, Hijmans M. Package ‘geosphere.’ Hijmans, RJ Williams, E Vennes, C Hijmans, MRJ. 2021.
8. Wickham H. ggplot2: Elegant Graphics for Data Analysis. Springer-Verlag New York; 2016.
