# Supplementary figures and images for: The natural and human-mediated expansion of a human-commensal lizard into the fringes of Southeast Asia
Source: BMC Ecol Evol. 2024 Feb 20;24:25. doi: 10.1186/s12862-024-02212-7 (PMC10880348; doi:10.1186/s12862-024-02212-7)

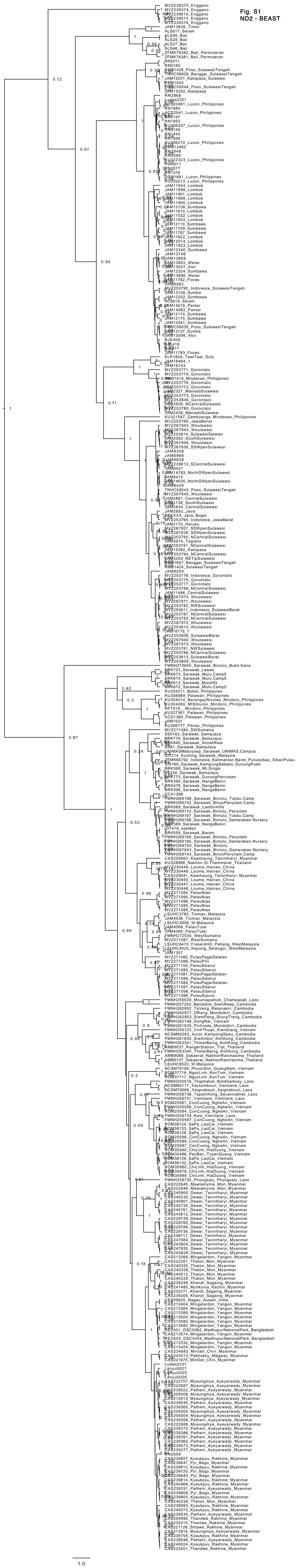

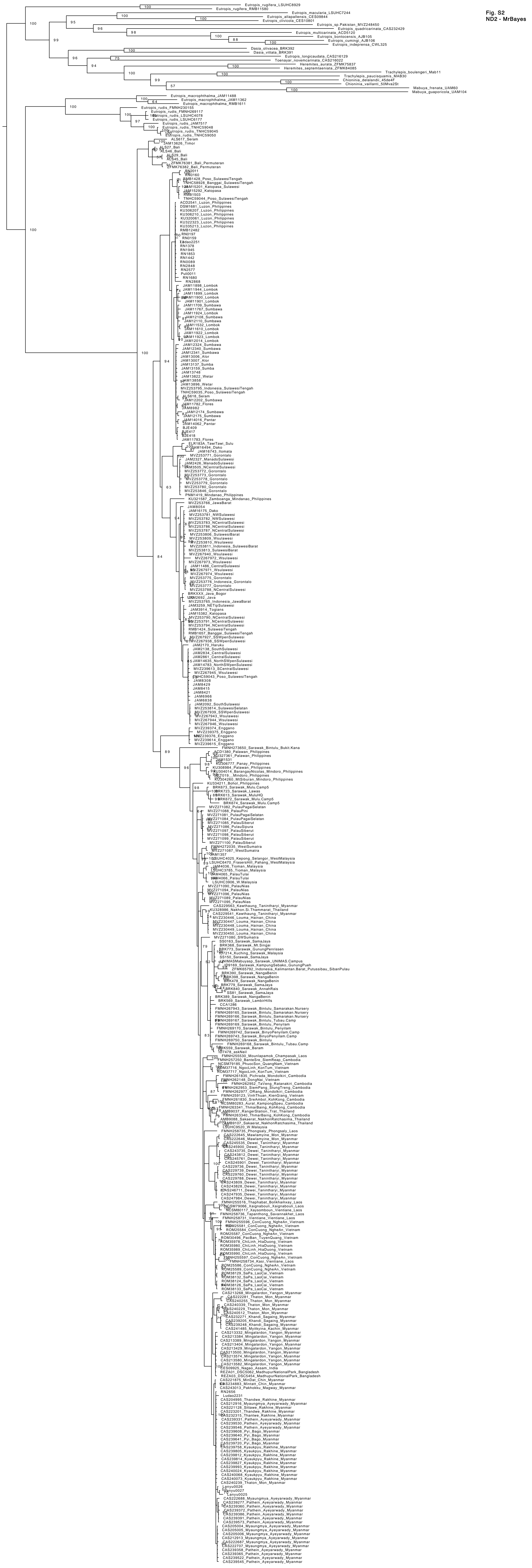

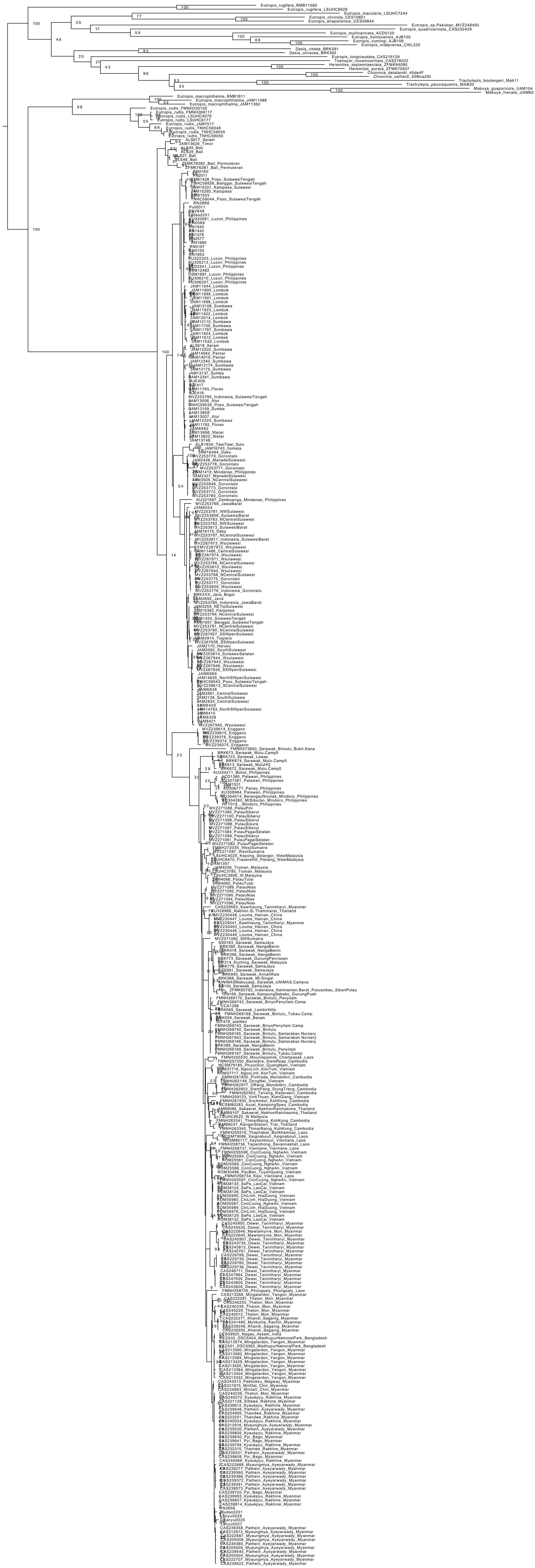

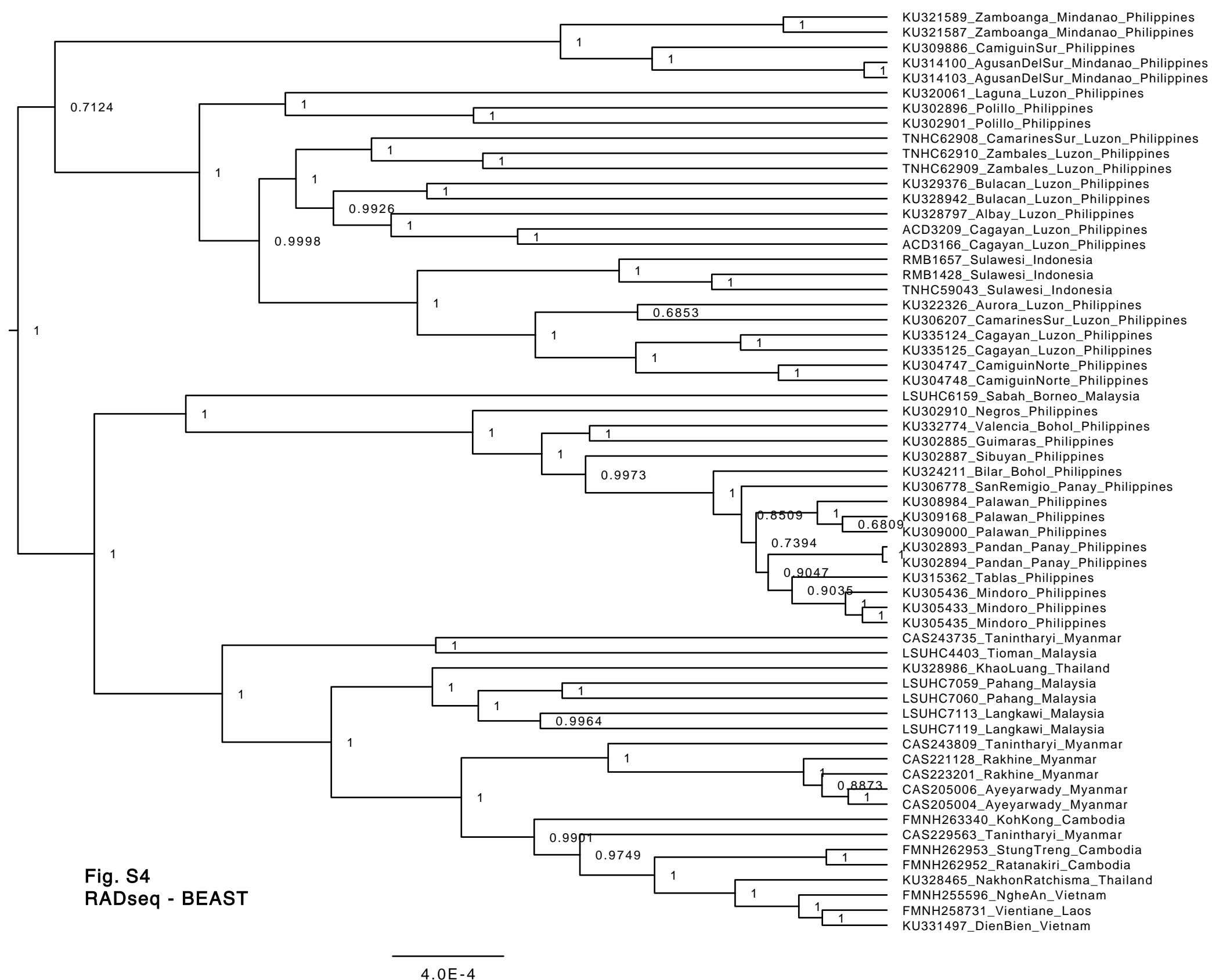

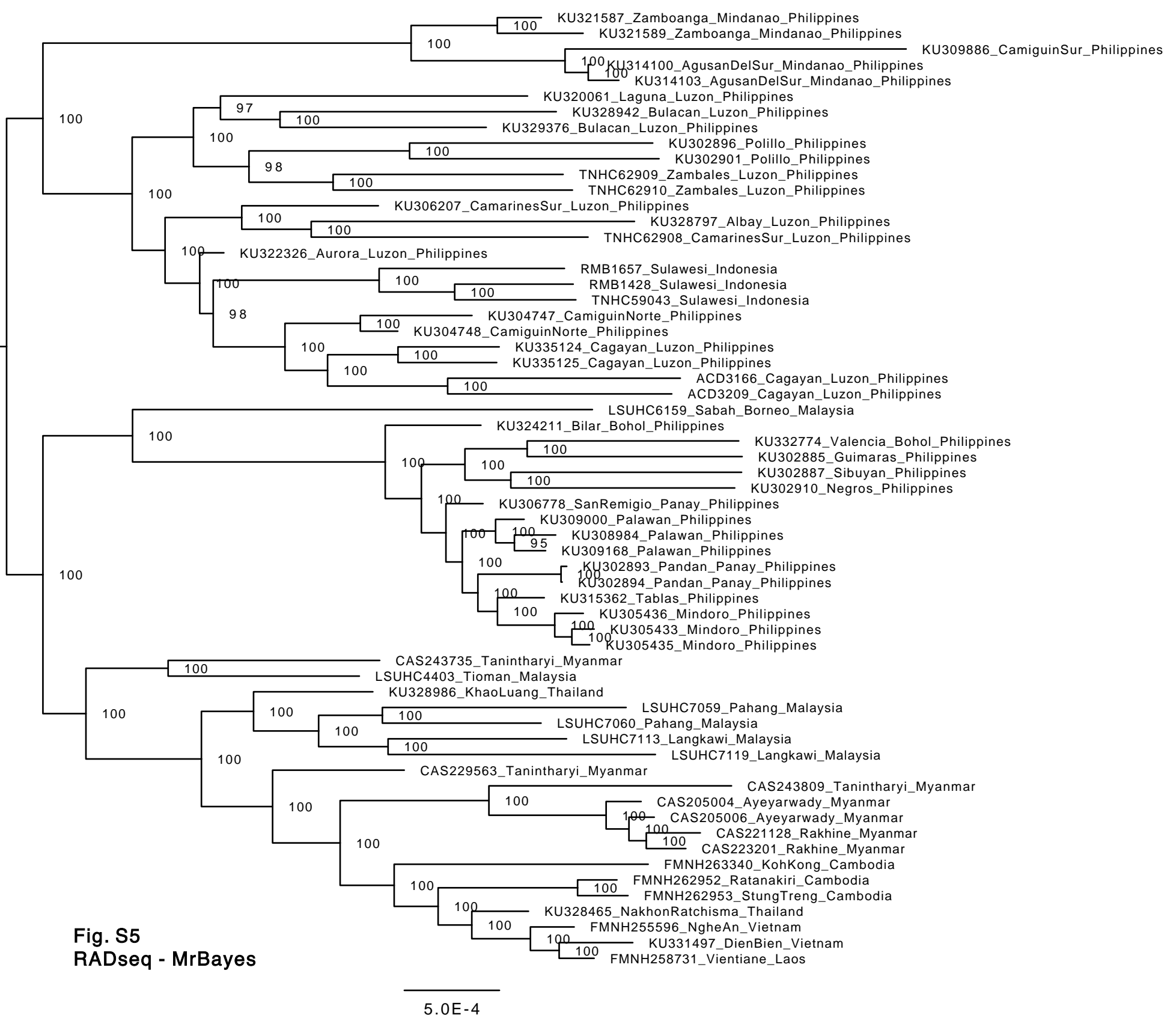

**Fig. S5**  
**RADseq - MrBayes**

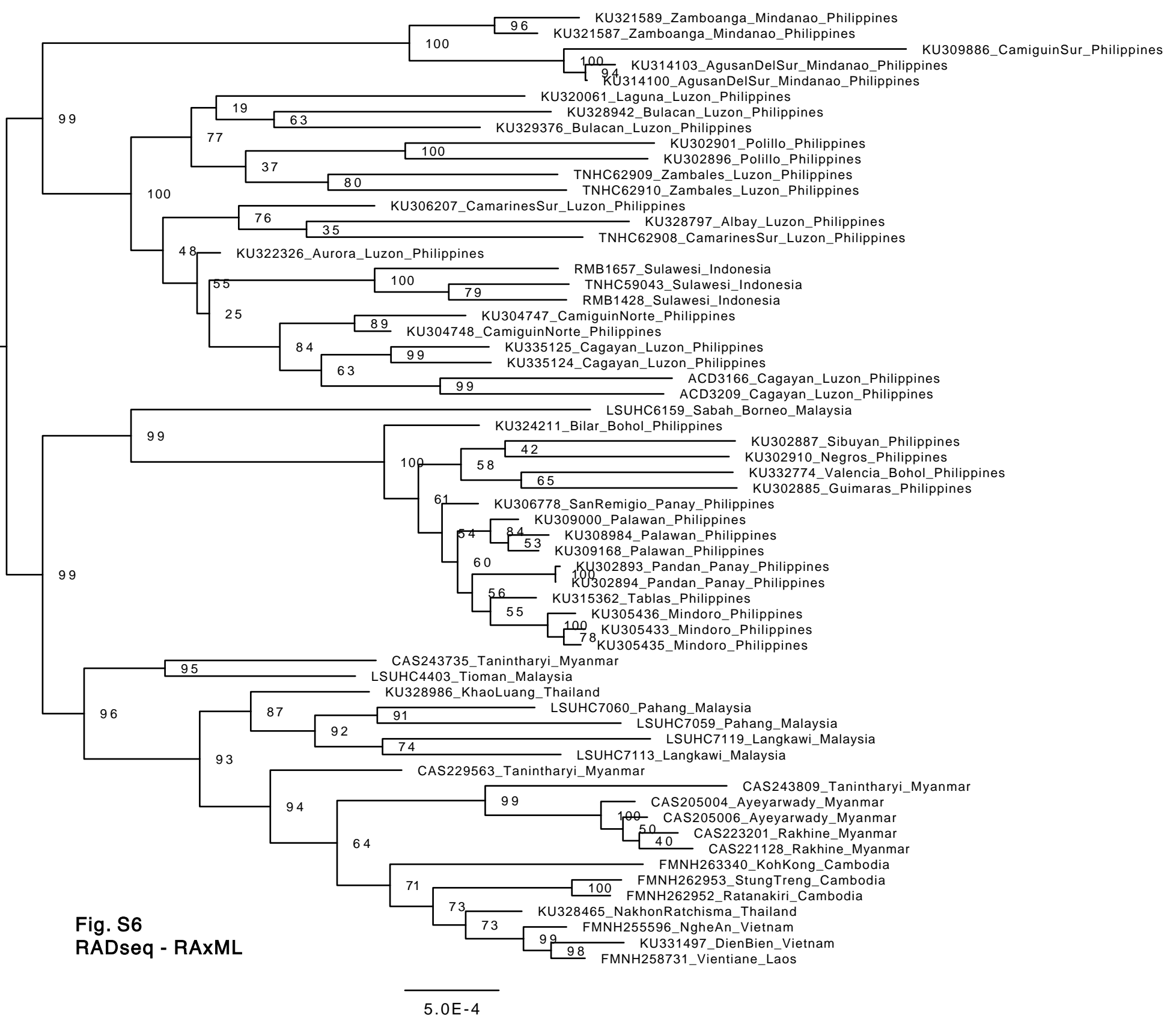

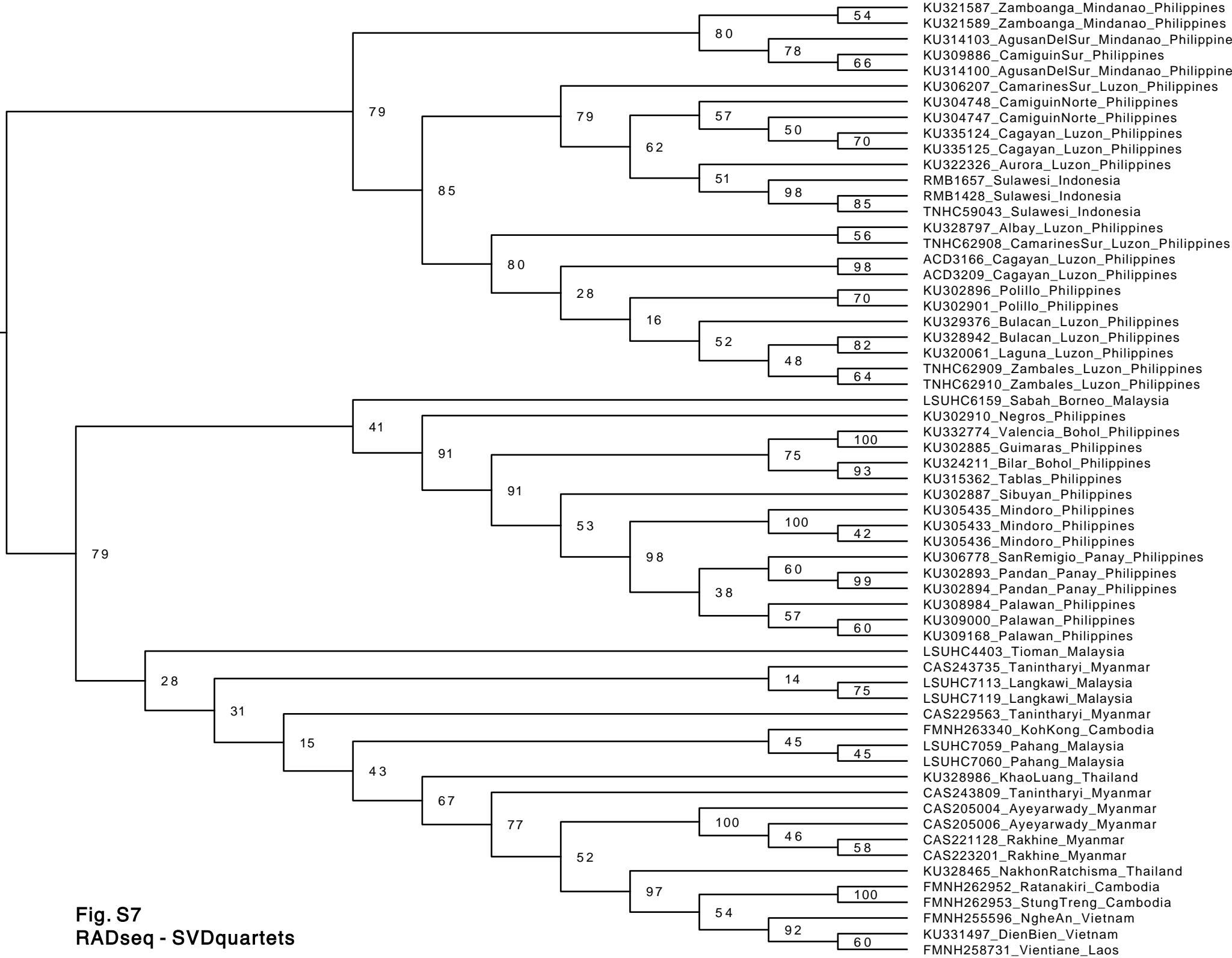

**Fig. S7**  
**RADseq - SVDquartets**

2.0

**Fig. S8**  
**Isolation By Distance Plot**

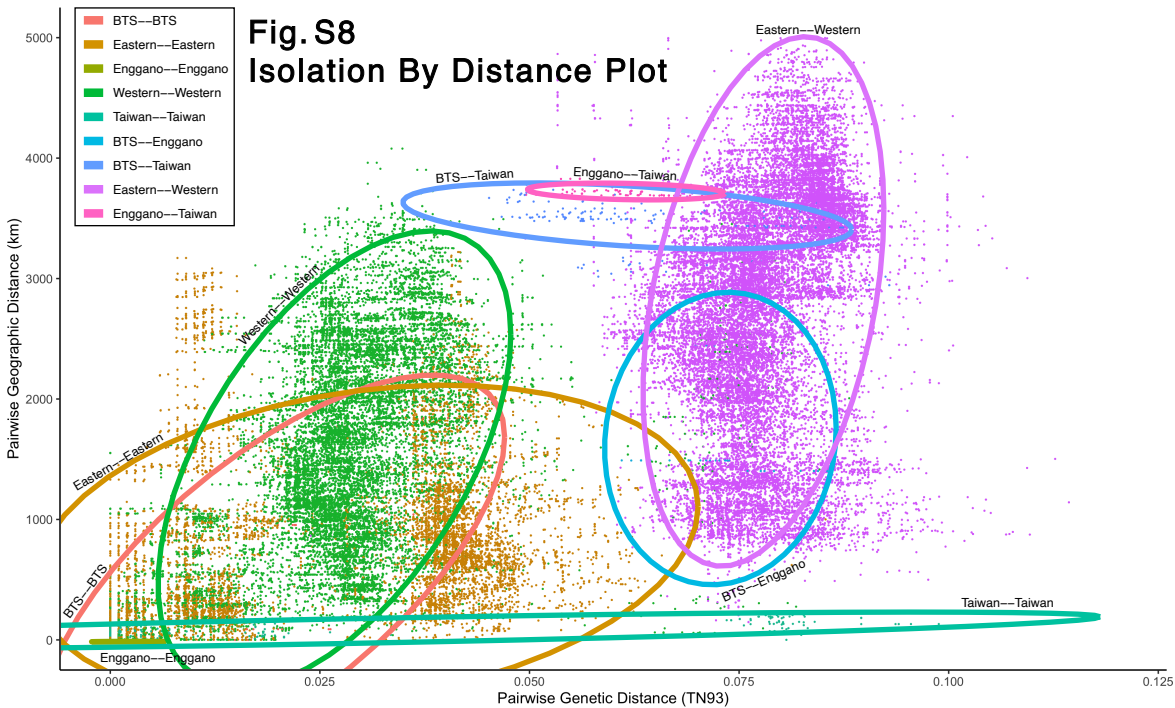

Supplement: Supplementary file 2 — Additional file 2: Figures S1-S8. [file 12862_2024_2212_MOESM2_ESM.pdf]
